# Gap-free genome assembly of anadromous *Coilia nasus*

**DOI:** 10.1038/s41597-023-02278-w

**Published:** 2023-06-06

**Authors:** Fengjiao Ma, Yinping Wang, Bixiu Su, Chenxi Zhao, Denghua Yin, Chunhai Chen, Yanping Yang, Chenhe Wang, Bei Luo, Hongqi Wang, Yanmin Deng, Pao Xu, Guojun Yin, Jianbo Jian, Kai Liu

**Affiliations:** 1 Wuxi Fisheries College, Nanjing Agricultural University, Wuxi, 214081 China; 2 Key Laboratory of Freshwater Fisheries and Germplasm Resources Utilization, Ministry of Agriculture and Rural Affairs, Freshwater Fisheries Research Center, Chinese Academy of Fishery Sciences, Wuxi, 214081 China; 3 BGI Genomics, BGI-Shenzhen, Shenzhen, 518083 China; 4 National Demonstration Center for Experimental Fisheries Science Education, Shanghai Ocean University, Shanghai, 201306 China

**Keywords:** Taxonomy, Freshwater ecology

## Abstract

The Chinese tapertail anchovy, *Coilia nasus*, is a socioeconomically important anadromous fish that migrates from near ocean waters to freshwater to spawn every spring. The analysis of genomic architecture and information of *C. nasus* were hindered by the previously released versions of reference genomes with gaps. Here, we report the assembly of a chromosome-level gap-free genome of *C. nasus* by incorporating high-coverage and accurate long-read sequence data with multiple assembly strategies. All 24 chromosomes were assembled without gaps, representing the highest completeness and assembly quality. We assembled the genome with a size of 851.67 Mb and used BUSCO to estimate the completeness of the assembly as 92.5%. Using a combination of *de novo* prediction, protein homology and RNA-seq annotation, 21,900 genes were functionally annotated, representing 99.68% of the total predicted protein-coding genes. The availability of gap-free reference genomes for *C. nasus* will provide the opportunity for understanding genome structure and function, and will also lay a solid foundation for further management and conservation of this important species.

## Abstract (summary)

**Table:** 

Measurement(s)	*Coilia nasus* • Gap-free genome assembly • sequence annotation
Technology Type(s)	DNBSEQ • Pacbio HiFi Sequencing • Nanopore Sequencing • Hi-C
Sample Characteristic - Organism	*Coilia nasus*
Sample Characteristic - Environment	freshwater
Sample Characteristic - Location	Taizhou City, Jiangsu Province, China

## Background & Summary

*Coilia nasus* (*C. nasus*, FishBase ID: 45335; Fig. 1A), also called Chinese tapertail anchovy, is a small to medium-sized migratory fish in the family Engraulidae, order Clupeiformes. Its native range extends from the coastal waters of China, Japan, and South Korea in the Northwest Pacific to interconnected freshwater tributaries (such as the Yellow River and Yangtze River). The middle and lower reaches of the Yangtze River and its affiliated lakes are the most important migration channels for *C. nasus*. During the spawning season, *C. nasus* migrates to river estuaries and further up to the middle and lower reaches of the Yangtze River and its conjoining lakes for spawning. Historically, some *C. nasus* reached Dongting Lake, over 1000 km upstream of the Yangtze River, to spawn^1^. After reproduction, these fish and their offspring return to the sea. Indeed, Yangtze *C. nasus* used to be one of the most important economic fish and was known as one of the “Three Yangtze Delicacies”, along with two other fishes (*Tenualosa reevesii* and *Takifugu fasciatus*). Due to its high social and nutritional value, Yangtze *C. nasus* has received overwhelming consumer demand in the last decade, thereby driving up the exploitation of the species. In 1973, the annual production of *C. nasus* from the Yangtze River reached 4142 tonnes^2^, and since then, it has been declining over the last few decades as a consequence of human influences, including overfishing, habitat degradation and other factors, reaching only 57.5 tonnes in 2012. Additionally, a strong response to stress (such as netting) would often cause tissue damage and death of *C. nasus*, which would reduce the survival rate and severely restrict the development of artificial breeding and large-scale cultivation of *C. nasus*^3,4^. As a result, *C. nasus* was listed as an endangered (EN) species in a 2018 Red List of Threatened Species report from the International Union for Conservation of Nature (IUCN) (www.iucnredlist.org). To protect this important resource in the Yangtze River, various strategies have been developed. For example, China has implemented a conservation policy for banning commercial fishing of Yangtze *C. nasus* since 2018 and implemented the longest and strictest 10-year fishing ban on the Yangtze River since 2021, which will be beneficial to the recovery of wild stocks of *C. nasus*. Therefore, it is urgent to assess the genetic background of *C. nasus* to further understand the diversity and population dynamics of this species and implement conservation efforts for wild populations.

**Fig. 1:**
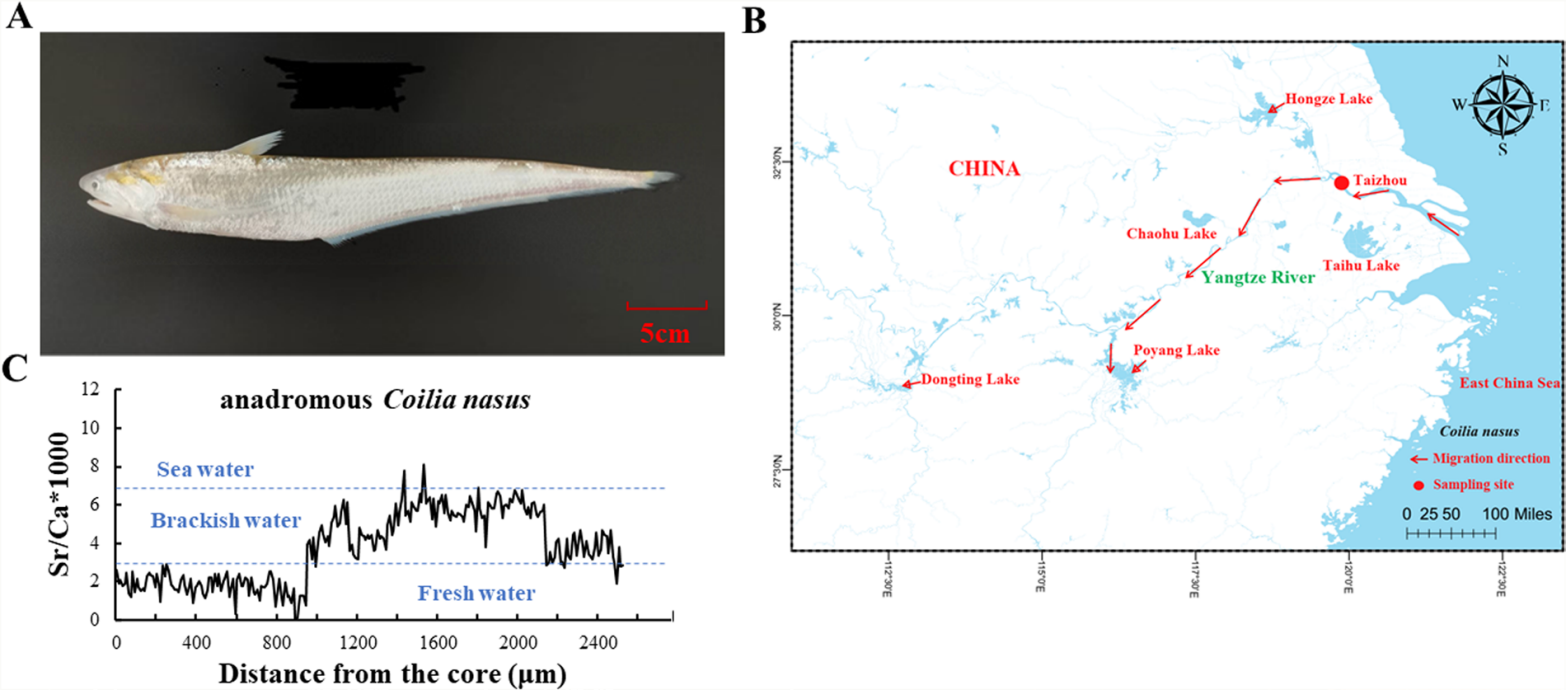
The sampling site and otolith profiles of *C. nasus*. (**A**) Morphological photograph of *C. nasus*. (**B**) Location of the sampling site (red dot) and migration direction (direction of the arrow) of *C. nasus*. (**C**) Otolith Sr: Ca profiles of *C. nasus* collected from the Taizhou section in the Yangtze River.

Many anadromous fish show complex migration patterns known as natal homing (migration to the place of natal origin) or natal river (migration to a general ‘home’ area, but not necessarily natal water), which requires remarkable precise orientation abilities^5^. In addition to the iconic example of salmonid migration, *C. nasus* may also exhibit natal homing behaviours^6^. If so, a question arises: how could they find their way back to their native rivers from these vast distances to locate their spawning grounds? This interesting question is divided into two subsequent questions. First, how does *C. nasus* orient itself at sea? Second, how does *C. nasus* locate its birth river? These questions have yet to be answered. Many different mechanisms have been demonstrated for orientation and navigation in some species with long-distance migration, including orientation using information from the sun, polarized light patterns, olfactory cues, and the Earth’s geomagnetic field^7–9^. During spawning migration in freshwater, anadromous fish rely primarily on the olfactory system to locate their spawning grounds^5^. Migratory fishes also respond to external triggering factors (such as water current, light, temperature, food availability, and upstream distance), which can trigger internal cues (such as circadian rhythm, hormones, and fat deposits) to drive migration and influence migration propensity^10^. For *C. nasus*, olfactory receptor genes have been identified at the transcript level, but the precise migration navigational mechanism is poorly understood^11^. In many migratory animals, migration exhibits a suite of traits with substantial phenotypic variability, which are most likely under genetic control and have been shown to be highly heritable^12,13^. Several studies have provided insight into the genetic mechanisms of migratory behaviour in animals that travel large distances and display precise homing ability^14–16^. Recent developments in genomics have resulted in a new and powerful molecular approach that could be used to study the genetics behind migratory behaviour. For example, many loci associated with migratory traits across many chromosomes in rainbow trout (*Oncorhynchus mykiss*) were found^17^. A region of the genome consisting of a large block of linkage disequilibrium in *O. mykiss* (on chromosome 5, or Omy5) is also reported to be closely associated with anadromy^18^. Based on the above studies, the identification of the chromosomal regions or genetic mechanisms by obtaining a gap-free reference genome will facilitate a deep understanding of the spawning migration behaviours of *C. nasus*.

Short-read sequencing technologies have led to a paradigm shift in biology over the last decade. Until recently, genomics has seen dramatic advances due to improvements in DNA sequencing technologies and assembly methodology, allowing the generation of more complete genome assemblies. The fast development of long-read sequencing technologies, such as the Pacific Biosciences (PacBio) HiFi and Oxford Nanopore Technology (ONT), has overcome early assembly limitations in continuity, correctness, and completeness, making it possible to understand the complexity and structure of genomes^19^. The long reads generated by PacBio HiFi and ONT are capable of resolving complex repetitive DNA regions on genome chromosomes, leading to a very contiguous assembly with higher mapping certainty^20,21^. As a complementary approach, high-throughput chromosome conformation capture (Hi-C) technology can capture chromatin three-dimensional structure information across the genome, and this spatial information can be used to assemble contigs and scaffolds as a chromosome-level^22^. Meanwhile, multiple assemblers that have been developed using different algorithms have provided an opportunity to generate high-quality assemblies and even achieve gap-free genomes.

Decoding complete genome sequence information is indispensable for the study of genomic variants and biological discoveries. In 2020, the high-quality reference genomes of cultivated *C. nasus* have been released^23^. However, the genome of the current version remained incomplete (87.1% complete), with many gaps. Gap-free genome assemblies are now a reality, allowing for nearly complete identification of genomic information, such as unique genes and structural variations (SVs)^24,25^. In recent years, gapless genomes of many species have been deciphered, such as Arabidopsis (*Arabidopsis thaliana*), rice (*Oryza sativa*), watermelon (*Citrullus lanatus*), banana (*Musa acuminata*), wild strawberry (*Fragaria vesca*), and humans^26–30^, but no gap-free genome assembly has been reported in *C. nasus*. In our study, we incorporated datasets of Pacific Biosciences (PacBio) HiFi reads, Nanopore Ultra-long reads, MGI short reads and Hi-C reads to assemble a gap-free genome of *C. nasus*, successfully bridging all the remaining assembly gaps across each chromosome in the currently available reference genomes. A gap-free genome assembly is not only essential to develop genomic research for *C. nasus* but is also a valuable resource for comparative genomics and evolutionary studies in *Coilia* fishes.

## Methods

### Sample collection, otolith validation and DNA extraction

A muscle sample was collected from a female *C. nasus* with a body weight of 211.8 g that was captured on 19 April 2021 in the Taizhou section of the Yangtze River, Jiangsu Province, China (32°12′N, 119°54′E), using a research boat from the Freshwater Fishery Research Center, Chinese Academy of Fishery Sciences (Fig. 1B). Sample collection was approved by the Department of Agriculture and Rural Affairs of Jiangsu Province, with the approval fishing licence code (Jiangsu) Scientific Fishing (2021) ZX-006 and −007. All specimen sampling was conducted in strict accordance with relevant guidelines and regulations established by the Animal Care and Use Committee of the Freshwater Fisheries Research Center, Chinese Academy of Fishery Sciences. According to a previously published study, the right sagittal otolith of *C. nasus* was used to confirm whether it was migratory using otolith fingerprint element technology^31^. As shown in Fig. 1C, the fluctuation patterns of Sr: Ca exhibit a life history of freshwater habitat, brackish water and seawater habitat, suggesting that the collected *C. nasus* specimen is typically anadromous. The muscle tissue below the dorsal fin was taken, quickly frozen in liquid nitrogen and stored at −80 °C for DNA sequencing for genome assembly.

### WGS library and PacBio library construction, sequencing and assembly

Long-read sequencing was performed using the PacBio Sequel-II platform, and the short but accurate reads from the MGISEQ platform were analysed for genome survey and evaluation of the assembly.

For the WGS library of short insert reads, genomic DNA was extracted from the muscle tissue by using MZ 1.3 (hypervariable minisatellite probe), as well as locus-specific minisatellite probes (g3, MS1 and MS43). Then these DNA samples were sheared into fragments between 50 and 800 bp using a Covaris E220 ultrasonicator (Covaris, Brighton, UK) according to the manufacturer’s recommendations. Between 300 and 400 bp were selected to construct a single-stranded circular DNA library and sequenced on an MGISEQ-2000 platform. A total of 86.07 Gb raw reads was generated (Table 1). Approximately 71.49 Gb of clean reads were retained after adapter sequence removal and low-quality read filtering by SOAPnuke v 2.0^32^ (parameters: -n 0.01 -l 20 -q 0.1 -i -Q 2 -G 2 -M 2 -A 0.5).

**Table 1:** Summary of the sequencing data obtained for *C. nasus* genome assembly.

Sequencing technology	Insert size	Raw data (Gb)	Clean data (Gb)	Average length (bp)
WGS	300–400 bp	86.07	71.49	150
PacBio HIFI	20 kb	610.48	36.75	15,396
Hi-C	300–400 bp	125.09	105.32	150
ONT	—	24.66	24.33	35,034

For the PacBio platform of long reads, genomic DNA was extracted from the same muscle tissue using a QIAGEN Blood & Cell Culture DNA Midi Kit following the manufacturer’s instructions (QIAGEN, Germany). After DNA preparation, two sequencing libraries were prepared according to the “Using SMRTbell Express Template Prep Kit 2.0 With Low DNA Input” protocol from PacBio and sequenced on a PacBio Sequel II SMRT cells in circular consensus sequence (CCS) mode with an insert size of approximately 20 kb (Pacific Biosciences, USA). After the removal of low-quality reads, a total of 36.75 Gb reads with a mean length of 15.4 kb were processed using the CCS version 4.0.0 (SMRTLink v 8.0.0) algorithm with the parameters “--minPasses 3--minPredictedAccuracy 0.99--minLength 500”.

With the HiFi reads of PacBio sequencing, the primary contigs were assembled using the default parameters of Hifiasm (v 0.15.1)^33^. Then the Purge Haplotigs program^34^ was used to remove redundant sequences with the parameters “-j 80 -s 80 -a 30”, which yielded a draft assembly with a size of approximately 850.52 Mb. The maximum contig size and N50 were 11.30 Mb and 0.97 Mb, respectively (Table 2).

**Table 2:** Statistics of *C. nasus* genome.

Statistical level	Parameter		
	Gap-free assembly	HiFi-contig	Published
Contig N50 (Mb)	35.42	0.97	1.6
Contig number (>100 bp)	117	2,465	1,327
Scaffold N50 (Mb)	—	—	2.1
Scaffold number (>100 bp)	—	—	727
Total length (Mb)	851.67	850.52	870.0
Maximum length (Mb)	45.20	11.30	12.0
Gap length (Mb)	0	0	0.49 (chromosomes only)
BUSCO Evaluation	C:92.5% [S:90.7%, D:1.8%], F:2.5%, M:5.0%, n:3640	C:91.5% [S:89.6%, D:1.9%], F:3.1%, M:5.4%, n:3640	C:87.1% [S:84.1%, D:4.6%], F:3.0%, M:9.9%, n:4584
Protein-coding genes	21,971	—	20,837

### Hi-C library preparation, sequencing and chromosome anchoring

To conduct the chromosome-level genome assembly, the draft genome contigs were anchored and oriented using the Hi-C data. In brief, muscle tissue (~1 g) of *C. nasus* was fixed with 1% formaldehyde for 10–30 min at room temperature to coagulate proteins that are involved in chromatin interaction in the genome. The restriction enzyme Mbo I (NEB, Ipswich, USA) was added to digest DNA, and fragments with flat or sticky ends were obtained. The processes of biotin marking, proximity ligations, crosslinking reversal, and DNA purification steps were used in previous studies^35^. The Hi-C library was made by capturing the biotin with magnetic beads and sequenced on the MGISEQ-2000 platform, and 125.09 Gb of Hi-C reads were generated (Table 1). A total of 105.32 Gb clean data were obtained from sequencing data using the software SOAPnuke v 2.0 with the parameters “-n 0.01 -l 20 -q 0.1 -i -Q 2 -G 2 -M 2 -A 0.5”.

**Fig. 2:**
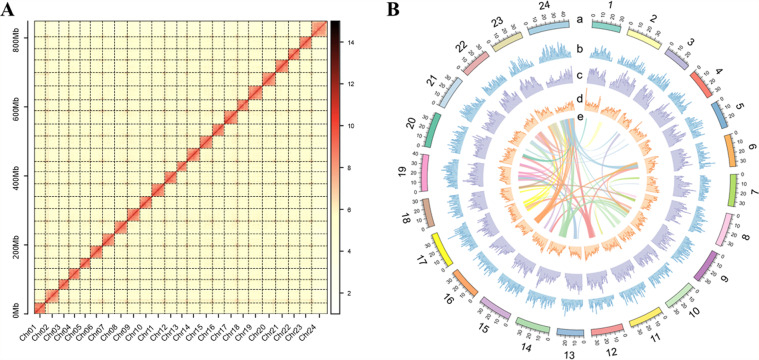
Hi-C chromatin interaction map and circos plot of the genome assembly. (**A**) Hi-C chromatin interaction map of the *C. nasus* assembly. (**B**) The circos plot of the *C. nasus* genome assembly. The rings from outside to inside indicate (a) chromosomes of the *Coilia nasus* genome, (b) gene density, (c) TE density, (d) GC density, and (e) paralogous genes on different chromosomes; b-d were drawn in 500-kb sliding windows.

The Hi-C sequencing data were aligned to the assembled contigs using BWA v 0.7.12^36^. We also utilized the juicer pipeline v 1.5 to remove the erroneous mappings (MAPQ = 0) and duplicated contigs to obtain the interaction matrix. Following this, approximately 193.87 Mb read pairs (~ 55.23%) were used to anchor the contigs into chromosomes with 3D-DNA pipeline v 180,922^37^. The 3D-DNA pipeline was used to remove select short contigs using default parameters. Scaffolds were manually checked and refined with JUICEBOX Assembly Tools (v 2.15.07)^38^. By using these Hi-C data, the assembled sequences were further anchored and oriented onto 24 chromosomes with a total length of 847.47 Mb, covering ~99.64% of the scaffold-level genome (Fig. 2A,B). The length of chromosomes ranged in size from 28.96 to 45.20 Mb (Table 3).

**Table 3:** Summary of assembled 24 chromosomes of *C. nasus*.

Chr ID	Chr Length (bp)	N%	GC%	Contig number
Chr01	31,585,711	0	43.91	1
Chr02	39,145,488	0	43.83	1
Chr03	28,961,466	0	43.95	1
Chr04	32,129,651	0	44.37	1
Chr05	29,751,362	0	44.08	1
Chr06	35,417,180	0	44.06	1
Chr07	35,246,941	0	44.33	1
Chr08	36,726,400	0	44.26	1
Chr09	36,500,450	0	44.23	1
Chr10	34,621,554	0	44.14	1
Chr11	37,229,399	0	44.29	1
Chr12	35,156,082	0	44.19	1
Chr13	29,427,943	0	44.05	1
Chr14	37,873,673	0	43.87	1
Chr15	36,085,597	0	44.33	1
Chr16	34,477,142	0	43.98	1
Chr17	38,792,572	0	43.86	1
Chr18	31,180,797	0	44.41	1
Chr19	40,394,057	0	44.18	1
Chr20	38,764,713	0	43.88	1
Chr21	36,550,783	0	44.16	1
Chr22	32,789,602	0	44.07	1
Chr23	34,608,544	0	44.08	1
Chr24	45,204,540	0	42.25	1

### Oxford Nanopore PromethION library preparation, sequencing and assembly

For ONT sequencing, genomic DNA was extracted using the CTAB method ( > 50 kb) with the SageHLS HMW library system (Sage Science) and was processed using the Ligation sequencing 1D kit (SQK-LSK109, Oxford Nanopore Technologies, Oxford, UK) according to the manufacturer’s instructions. Then, the ONT library was prepared. The genome was sequenced on the Nanopore PromethION platform (Oxford Nanopore Technologies) at the Genome Center of Grandomics (Wuhan, China). After filtering with length < 5 kb and quality value < 7, a total of 24.33 Gb of ONT long reads were generated, the N50 of ONT long reads was 47.11 kb, and the longest reads were 462.45 kb. The ultra-long ONT reads were corrected to improve the final consensus assembly by NECAT (https://www.nature.com/articles/s41467-020-20236-7) with the following parameters: ‘OVLP_FAST_OPTIONS = -n 500 -z 20 -b 2000 -e 0.5 -j 0 -u 1 -a 1000’ and ‘CNS_FAST_OPTIONS = -a 2000 -x 4 -y 12 -l 1000 -e 0.5 -p 0.8 -u 0’. The consensus ONT ultra-long reads were used to fill gaps of the above assembly by running three iterations of LR_Gapcloser (v1.0)^39^ and TGS-GapCloser (v 1.0.1)^40^ with the parameter “--min_match 2000”. The gap-free level was reached after three rounds of gap filling. With all these processes, we generated a genome assembly of *C. nasus*, where the genome size was approximately 851.67 Mb and N50 was 35.42 Mb (Table 2).

### Repetitive sequence annotation

A combined strategy based on *de novo* searches and homologue alignments was used to annotate whole-genome repeat elements. A *de novo* repetitive element database was identified by Repeat Modeler v 1.0.4^41^ and long terminal repeats were annotated by LTR-FINDER v 1.0.7^42^. For homologue prediction, DNA and protein transposable elements (TEs) were detected by RepeatMasker (v 4.0.7)^43^ and RepeatProteinMasker (v 4.0.7)^44^, respectively, based on the Repbase database. Tandem repeats were performed by Tandem Repeat Finder v 4.10.0^45^. The combination of Repbase and our *de novo* TE library revealed that 38.26% of the assembled *C. nasus* genome was annotated as repetitive elements, of which short interspersed nuclear elements (SINEs) and long terminal repeats (LTRs) accounted for 0.44% and 8.86% of the whole genome, respectively, and long interspersed nuclear elements (LINEs) accounted for 9.98% (Table 4).

**Table 4:** Summary statistics of repetitive sequences annotation in *C. nasus* genome.

Biological classification	Repbase TEs		TE proteins		*De novo*		Combined TEs	
	Repeat size (bp)	Percentage of genome (%)	Repeat size (bp)	Percentage of genome (%)	Repeat size (bp)	Percentage of genome (%)	Repeat size (bp)	Percentage of genome (%)
DNA	153,326,315	18.00	559,532	0.07	220,466,146	25.89	254,472,881	29.88
LINE	29,368,185	3.45	10,548,699	1.24	66,307,405	7.79	84,986,643	9.98
SINE	1,945,034	0.23	0	0	1,870,905	0.22	3,717,061	0.44
LTR	31,939,152	3.75	7,598,703	0.89	50,120,328	5.88	75,474,097	8.86
Other	15,618	0.002	0	0	0	0	15,618	0.002
Unknown	0	0	0	0	11,896,219	1.40	11,896,219	1.40
Total	188,282,612	22.11	18,704,541	2.20	300,973,021	35.34	325,813,602	38.26

### Protein-coding gene annotation

**Fig. 3:**
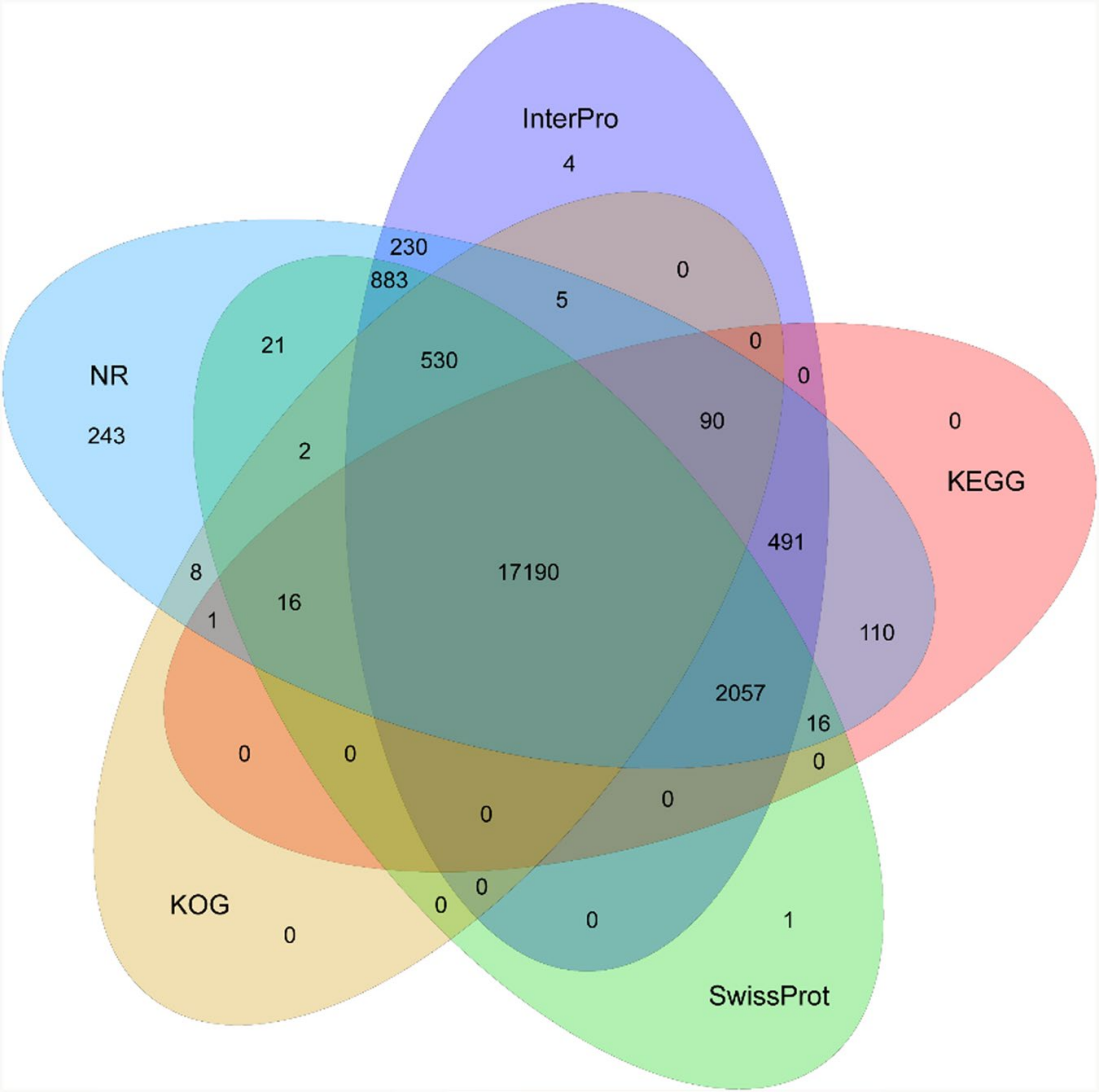
Venn diagram of functional annotation of the *C. nasus* protein-coding genes. The Venn diagram shows the shared and unique annotations among InterPro, KEGG, KOG, NR and SwissProt.

To obtain protein-coding genes, we employed *de novo* prediction, homology-based annotation and RNA-Seq assisted prediction. For *de novo* prediction, gene models of *C. nasus* were predicted by Augustus (v 3.2.1)^46^ with default parameters. For homology-based prediction, protein sequences of six representative teleosts, including *Clupea harengus* (GCF_900700415.2.), *Danio rerio* (GCF_000002035.6), *Denticeps clupeoides* (GCF_900700375.1), *Electrophorus electricus* (GCF_013358815.1), *Oncorhynchus mykiss* (GCF_013265735.2) and *Sardina pilchardus* (GCA_900499035.1), were downloaded from the National Center for Biotechnology Information (NCBI). GeMoMa (v1.8) was used to search coding structures based on transcriptome data and homologous proteins^47^. For the transcriptome-based annotation, pooled RNA-seq reads from the liver, brain, and stomach were mapped onto the *C. nasus* genome by using Hisat2 (v 2.1.0)^48^ with the following parameters:--sensitive--no-discordant--no-mixed -I 1 -X 1000--max-intronlen 1000000. The aligned reads were assembled using Stringtie (v 1.3.5)^49^ with the following parameters: -f 0.3 -j 3 -c 5 -g 100 -s 10000. Subsequently, TransDecoder (v 5.5.0; https://github.com/TransDecoder/TransDecoder) was used to identify the coding sequence with default parameters. The abovementioned transcriptome data and homologous proteins were merged by GeMoMa v1.8 software. A total of 21,971 protein-coding genes with a mean length of 23,357 bp were predicted (close to the 21,469 of *Danio rerio*; Table 5). The final gene sets were functionally annotated by aligning the gene sequences to KEGG (Kyoto Encyclopedia of Genes and Genomes, http://www.genome.jp/kegg/), Swiss-Prot (http://www.gpmaw.com/html/swiss-prot.html), TrEMBL (http://www.uniprot.org), KOG^50^, and NR (NCBI nonredundant protein) databases using BLASTp v 2.2.26^51^ with an E-value threshold of 1E-5. The protein domains and motifs were annotated using InterProScan^52^. GO Ontology (GO)^53^ was obtained from the InterProSca results in this study. Approximately 99.68% (21,900 genes) of the total predicted genes were successfully annotated by at least one database (Fig. 3 and Table 6). Of these functional proteins, 17,190 genes (~78.24%) were supported by all five databases.

**Table 5:** Statistics of predicted protein-coding genes in the *C. nasus* genome.

Parameter	Gene set	Number	Average gene length (bp)	Average CDS length (bp)	Average exon per gene	Average exon length (bp)	Average intron length (bp)
*De novo*	Augustus	29,631	12,338	1,183	6	197	2,226
Homolog	*Clupea harengus*	25,361	23,222	1,719	12	494	1,669
	*Danio rerio*	21,469	24,297	1,749	11	500	1,777
	*Denticeps clupeoides*	20,773	25,776	1,789	12	490	1,780
	*Electrophorus electricus*	19,846	25,817	1,770	12	488	1,783
	*Oncorhynchus mykiss*	22,628	25,416	1,760	12	493	1,784
	*Sardina pilchardus*	27,993	15,027	1,181	8	609	1,372
RNA-seq	transcript	21,700	22,758	1,650	11	308	1,919
Final	—	21,971	23,357	1,673	11	520	1,660

**Table 6:** Statistics of functional annotation of *C. nasus*.

Data type	Total	Nr	Swissprot	KEGG	KOG	TrEMBL	Interpro	GO	Overall
Number	21,971	21,893	20,716	19,971	17,842	21,889	21,480	15,797	21,900
Percentage	—	99.64%	94.29%	90.90%	81.21%	99.63%	97.77%	71.90%	99.68%

## Data Records

The sequencing dataset and genome assembly of *C. nasus* have been deposited in the Sequence Read Archive (SRA) under project number SRP405363^54^. DNA sequencing data from the WGS library were deposited in the SRA at SRR22102323^55^. DNA sequencing data from the ONT library were deposited in the SRA at SRR22102324^56^. DNA sequencing data from the Hi-C library were deposited in the SRA at SRR22102325^57^. DNA sequencing data from the PacBio HiFi library were deposited in the SRA at SRR22102326^58^. This Whole Genome Shotgun project was deposited at GenBank under accession JAPTFL000000000^59^. Moreover, files of the assembled genome, gene structure annotation and repeat prediction annotation of *C. nasus* were deposited in Figshare database under DOI code^60^.

## Technical Validation

### Evaluation of the genome assembly

The paired-end short reads (including DNA and RNA sequencing) were mapped to the assembled genome using BWA software, and the results showed that 98.06% and 96.21% of the reads could be mapped, respectively. Furthermore, the HiFi sequencing data was mapped to the assembled genome using Minimap2, with a mapping rate of 99.82%^61^. In terms of some assembled metrics, such as contig lengths, gap number and BUSCO completeness, our new genome assembly showed a great improvement compared to the previously reported *C. nasus* genome. By comparing previously published assembly data, our gap-free genome assembly increased the contiguity metrics by contig N50^23^. Among the published genomes in Clupeiformes, the assembly in this study had the longest contig N50 length and was the first gap-free genome, suggesting that our *C. nasus* genome was of high quality (Table 2).

The completeness of the assembled genome sequence was evaluated using Benchmarking Universal Single-Copy Orthologs (BUSCO, v 5.1.0). The BUSCO analysis based on the actinopterygii_odb10 database showed that 92.5% of the expected actinopterygii_odb10 genes (single-copy genes: 90.7% and duplicated genes: 1.8%) were identified as complete, and 2.5% fragmented genes were found in the genome assembly. However, 5% were missing from our *C. nasus* genome. Nevertheless, the complete evaluation of the *C. nasus* genome was superior to other current public cetacean genomes.

### Evaluation of the gene annotation

With this gap-free reference genome, we identified approximately 325.81 Mb repetitive sequences of the assembled *C. nasus* genome, accounting for 38.26% of the total genome sequences. The repetitive elements in the *C. nasus* genome sequences were masked, and the repeat-masked genome was used for the gene prediction (Tables 7, 8).

**Table 7:** Summary of transposon element families in *C. nasus* based on various methods.

Type	Repeat Length(bp)	% of genome
Trf	142,366,862	16.72
Repeatmasker	188,282,612	22.11
Proteinmask	18,704,541	2.20
*De novo*	327,062,561	38.40
Total	384,689,436	45.17

**Table 8:** Statistics of classified repeat in the *C. nasus*.

	Type	Length(bp)	% of genome
Retro	LTR/Copia	1,756,909	0.21
	LTR/Gypsy	24,610,355	2.89
	LTR/Other	51,558,579	6.05
	SINE	3,656,961	0.43
	LINE	84,946,945	9.97
	Other	0	0
DNA	EnSpm	157,557,719	18.50
	Harbinger	6,647,946	0.78
	hAT	51,565,767	6.05
	Helitron	24,163,449	2.84
	Mariner	2,542,498	0.30
	MuDR	1,509,936	0.18
	P	759,151	0.09
	Other	147,606,832	17.33
Other	—	26,095,309	3.06
Unknown	—	11,896,219	1.40
Total	—	351,904,065	41.32

**Fig. 4:**
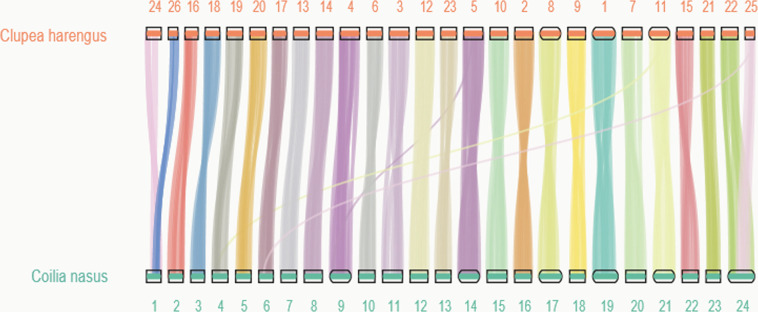
Chromosome comparison of *C. nasus* to *Clupea harengus* using protein-coding gene synteny. The chromosome ID of *C. nasus* was sorted by sequence length.

We also performed BUSCO analysis with the actinopterygii_odb10 database to assess the completeness of the coding sequences for *C. nasus*. The results showed a total of 21,971 protein-coding genes, and each gene had an average number of 11 exons (Table 9). Approximately 99.68% (21,900 genes) of the total predicted genes were assigned with at least functional annotation, showing a more complete annotation. Furthermore, we compared the conservation synteny between *C. nasus* and *C. harengus* to validate the chromosome assembly^62^. We observed highly conserved synteny and strict correspondence of chromosome assignment (Fig. 4).

**Table 9:** The evidence supporting gene models of the *C. nasus* genome.

	> = 30% overlap		> = 50% overlap		> = 80% overlap	
	No.	Ratio (%)	No.	Ratio (%)	No.	Ratio (%)
R (single)	0	0	0	0	0	0
H (single)	688	3.13	782	3.56	1,246	5.67
H (more)	1,602	7.29	2,202	10.02	4,213	19.18
P (single)	0	0	0	0	0	0
P (more)	0	0	0	0	0	0
HR	2,754	12.53	3,987	18.15	7,335	33.38
PR	0	0	0	0	0	0
PH	5,160	23.49	4,784	21.77	3,379	15.38
PHR	11,767	53.56	10,216	46.5	5,798	26.39
Total	21,971	100	21,971	100	21,971	100

R: refers to evidences from transcriptomic prediction; P: refers to evidences from *de novo* prediction; H: refers to evidences from homologous prediction; single: refers to one type of evidence; more: refers to more than one evidence; “> = 30% overlap” refers to 30% of the query sequences are aligned onto target sequences; “> = 50% overlap” refers to 50% of the query sequences are aligned onto target sequences; “> = 80% overlap” refers to 80% of the query sequences are aligned onto target sequences.

## Data Availability

No specific code was developed for this work. The data analyses were performed according to the manuals and protocols provided by the developers of the corresponding bioinformatics tools in the methods.
